# Phenotype, genotype, and management of congenital fibrosis of extraocular muscles type 1 in 16 Chinese families

**DOI:** 10.1007/s00417-022-05830-3

**Published:** 2022-09-23

**Authors:** Moxin Chen, Rui Huang, Yingjie Zhang, Deyi Jasmine Zhu, Qin Shu, Pengcheng Xun, Jing Zhang, Ping Gu, Lin Li

**Affiliations:** 1 Department of Ophthalmology, Shanghai Ninth People’s Hospital, School of Medicine, Shanghai Jiao Tong University, Shanghai 639 Zhizaoju Road, Huangpu District, 200011 Shanghai, China; 2 Shanghai Key Laboratory of Orbital Diseases and Ocular Oncology, Shanghai, China; 3 Brooks School, North Andover, MA USA; 4 Department of Global Value Access and Outcomes, Atara Biotherapeutics, Thousand Oaks, CA USA

**Keywords:** CFEOM1, *KIF21A*, Ptosis, Hotspot mutations

## Abstract

**Purpose:**

Congenital fibrosis of extraocular muscles type 1 (CFEOM1), a classical subtype of CFEOM, is characterized by restrictive ophthalmoplegia and ptosis. It is mainly caused by aberrant neural innervation of the extraocular muscles. This study aimed to investigate the genetic characteristics and clinical manifestations of CFEOM1 in Chinese families.

**Methods:**

The clinical data, including ocular examinations, magnetic resonance imaging (MRI), and surgical procedures of affected individuals from 16 Chinese CFEOM1 families, were collected. The genomic DNA of 16 probands and their family members were sequenced for causative *KIF21A* gene mutations. Linkage analysis using microsatellite markers across *KIF21A* was also conducted.

**Results:**

Affected individuals were presented with bilateral non-progressive ptosis, restricted horizontal eye movement, fixed infraduction of both eyes, compensatory chin-up head position, and neuromuscular abnormalities. Three heterozygous *KIF21A* mutations, c.2860C > T (p.R954W) (in eight families), c.2861G > T (p.R954L) (in two families), and c.2861G > A (p.R954Q) (in two families) were identified, which implied that hotspot mutations were common in Chinese CFEOM1 families. Germline Mosaicism was likely to be the cause of affected individuals with asymptomatic parents without *KIF21A* mutations presented in the eight families. Two affected individuals underwent modified levator muscle complex suspension surgery and achieved a good result without any complications.

**Conclusion:**

Instead of evaluating the whole CFEOM1 gene variant, hotspot mutations could be given priority for screening. The occurrence of germline mosaicism has to be taken into account in genetic counseling. Patients with CFEOM1 who have ptosis may benefit from an innovative surgical procedure called modified levator muscle complex suspension.

**Supplementary Information:**

The online version contains supplementary material available at 10.1007/s00417-022-05830-3.

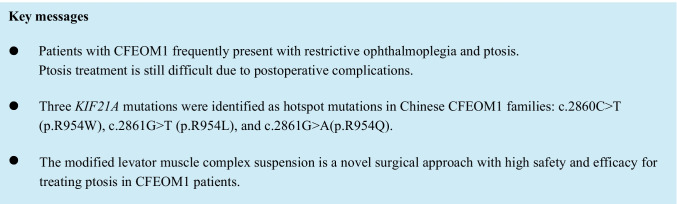



## Introduction

Congenital fibrosis of extraocular muscles (CFEOM) is a hereditary monogenic extraocular muscle disorder with a prevalence of 1/250 000–1/230 000 [1, 2]. CFEOM is characterized by impaired eye movements and ptosis, and is classified into three main subtypes, CFEOM type 1 (CFEOM1), CFEOM type 2 (CFEOM2), and CFEOM type 3 (CFEOM3) [3], according to the pattern of inheritance and clinical manifestations.

CFEOM1 is the most common subtype of CFEOM, inherited in an autosomal dominant pattern, and caused by heterozygous mutations in the kinesin family member 21A (*KIF21A*) gene located in chromosome 12q12 [4]. Individuals with CFEOM1 typically present with fixed infraduction, variably severe ophthalmoplegia, and ptosis, without pupillary involvement [5, 6].

CFEOM2 has autosomal recessive inheritance and is caused by homozygous mutations in paired like homeobox 2A (*PHOX2A*) gene located in chromosome 11q13 [7]. The phenotype of CFEOM2 is distinctively characterized as bilateral large-angle exotropia, ptosis, poorly reactive pupils, and sporadically accompanied by retinal dystrophy [7, 8].

CFEOM3 is autosomal dominant inherited and is caused by either *KIF21A* gene mutations [9–12] or three different tubulins: tubulin beta 3 class III (*TUBB3*) located in chromosome 16q24, tubulin beta 2B class IIb (*TUBB2B*) located in chromosome 6p25, and tubulin alpha 1a (*TUBA1A*) located in chromosome 12q13. Unilateral and asymmetric ptosis and oculomotor defect are often present in individuals with CFEOM3 [13]. Individuals with CFEOM3 may have other developmental abnormalities. CFEOM3 with ulnar hand anomalies is defined as Tukel syndrome [14].

In clinical practice, screening *KIF21A* gene mutation and investigating genetic etiology for CFEOM patients are helpful to subtype diagnosis. In previous studies, twelve different missense mutations and one deletion mutation were reported in CFEOM1 families [15–17]. Understanding the mutational spectrum of CFEOM patients could benefit mutation screening and guide the directions of basic research in the future.

Ptosis is a common symptom in CFEOM1 individuals; thus, the patients may have a compensatory chin up head positions and are more likely to develop amblyopia [18]. CFEOM1 patients may have a higher risk of exposure keratitis after ptosis surgery [19] due to eye infraduction and poor Bell’s reflex. Therefore, finding a safe ptosis surgical procedure for CFEOM1 patients is of great importance. Herein, we reported a novel modified levator muscle complex suspension procedure, which has been shown great effectiveness in treating other congenital severe ptosis [20], to treat CFEOM1 patients with ptosis.

In this study, we aimed to investigate the genetic characteristics and clinical manifestations of individuals from 16 Chinese CFEOM1 families. We also summarized the genetic profiles and surgical procedures of CFEOM1 families in our results with previous studies together, to provide comprehensive and up-to-date information for CFEOM1 diagnosis and treatment.

## Materials and methods

### Study design, setting, and participants

A total of 35 patients (18 males and 17 females) from 16 families were recruited in Shanghai Ninth People’s Hospital from March 2016 to January 2022. A detailed chart review of each individual’s medical record was conducted retrospectively from February 2022 to April 2022. Written informed consent was obtained from each participant, or legal guardians when the participant is under the age of 18. After providing informed consent, affected individuals underwent ophthalmology examinations, and surgeries if necessary. Affected individuals and their relatives provided blood samples for mutation screening and haplotype analysis if available. This study protocol was reviewed and approved by the Ethics Committee of Shanghai Ninth Hospital, School of Medicine, Shanghai Jiao Tong University (SH9H-2021-T89-1).

### Clinical analysis

Clinical selection criteria were congenital non-progressive restrictive ophthalmoplegia, with or without ptosis. Each patient underwent ophthalmological and magnetic resonance imaging (MRI) examinations (Table 1). The presence, type, and amount of strabismus were measured by prism plus cover testing. The presence of ptosis was defined as a margin reflex distance of 2 mm or below in either or both eyes. Surgical procedures (including strabismus and ptosis surgeries) were customized based on the severity of symptoms and individual informed preference (Table 2). Full correction of strabismus was defined as orthotropia in primary position, and microtropia was defined as a deviation of greater than 5 PD, but less than 10 PD in primary position. Full correction of ptosis was defined as margin reflex distance of 3 mm or above, and under-correction was defined as margin reflex distance under 3 mm. After surgery, there was an average follow-up time of 3.5 years.

**Table 1:** Ophthalmologic examinations and genetic screening of probands from sixteen CFEOM1 families^a^

Subject	Age	Gender	Corrected visual acuity	Fixed infraduction	Restricted horizontal eye movement	Prism plus cover testing (△)	Ptosis	Eye movement	Compensatory head position	Titmus stereopsis	*KIF21A* mutation
01	17	M	R: 20/100 L: 20/63	+	ET	120	+	Restrictive	Chin up head position	nil	c.2861G > T (p.R954L)
02	29	M	R: 20/50 L: 20/32	+	ET	25–30	+	Restrictive	Chin up head position	nil	c.2860C > T (p.R954W)
03	2	M	NA	+	ET	NA	+	Restrictive	Chin up head position	NA	c.2860C > T (p.R954W)
04	3	F	R: 20/20 L: 20/25	+	ET	90	+	Restrictive	Chin up head position	NA	c.2860C > T (p.R954W)
05	6	M	R: 20/25 L: 20/25	+	ET	NA	+	Restrictive	Chin up head position	NA	c.2860C > T (p.R954W)
06	5	M	R: 20/50 L: 20/50	+	ET	NA	+	Restrictive	Chin up head position	nil	c.2860C > T (p.R954W)
07	6	M	R: 20/50 L: 20/63	+	XT	50	+	Restrictive	Chin up head position	NA	c.2860C > T (p.R954W)
08	5	F	R: 20/50 L: 20/100	+	XT	40–50	+	Restrictive	Chin up head position	200 s	c.2860C > T (p.R954W)
09	26	M	R: 20/32 L: 20/25	+	ET	15–20	+	Restrictive	Chin up head position	NA	c.2860C > T (p.R954W)
10	1	F	NA	+	ET	NA	NA	Restrictive	Chin up head position	NA	c.2861G > A (p.R954Q)
11	27	F	NA	+	XT	90–100	+	Restrictive	Chin up head position	NA	c.2861G > A (p.R954Q)
12	21	F	NA	-	XT	30	-	Restrictive	None^b^	NA	c.2861G > T (p.R954L)
13	35	F	NA	-	XT	20	+	Restrictive	Chin up head position	NA	didn’t find
14	27	F	R: 20/200 L:20/32	+	ET	50–60	+	Restrictive	Chin up head position	200 s	didn’t find
15	1	F	NA	+	ET	60	+	Restrictive	Chin up head position	NA	didn’t find
16	13	F	R: 20/800 L: 20/40	+	XT	90–110	+	Restrictive	Chin up head position	nil	didn’t find

M, male. F, female. ET, esotropia. XT, exotropia. NA, not applicable. nil, no stereopsis

^a^The information above is related to the probands of each family in this study

^b^This patient had ptosis surgery before and didn’t have ptosis and compensatory head position when she visited our department of ophthalmology

**Table 2:** Strabismus and ptosis surgeries conducted in CFEOM1 patients^a^

Subject	Surgery type	Surgical Procedure	Surgical outcome
01	Strabismus	Bilateral IRM recession (8 mm), with IRM nasal transposition	Orthotropia in primary position
04	Strabismus	Bilateral LRM recession (6 mm), bilateral IRM recession (5 mm), and suspension (3 mm)	Orthotropia in primary position
07	Strabismus	Bilateral MRM recession (8 mm) and suspension (2 mm), bilateral IRM recession (6 mm), and suspension (2 mm)	Microtropia in primary position
11	Strabismus	Bilateral MRM recession (8 mm) and suspension (2 mm), bilateral IRM recession (5 mm), suspension (2 mm), and transition (8 mm)	Microtropia in primary position
15	Strabismus	Bilateral LRM recession (6 mm), and bilateral IRM recession (8 mm)	Microtropia in primary position
16	Strabismus	Bilateral LRM resection (6 mm)	Microtropia in primary position
02	Ptosis	Levator palpebrae superioris muscle shortening	Undercorrection
08	Ptosis	Modified combined fascia sheath and levator muscle complex suspension with Müller muscle preservation	Full correction
12	Ptosis	Levator palpebrae superioris muscle shortening	Undercorrection
15	Ptosis	Modified combined fascia sheath and levator muscle complex suspension with Müller muscle preservation	Full correction
16	Ptosis	Frontal muscle flap suspension	Undercorrection

NA, not applicable. IRM, inferior rectus muscle. LRM, lateral rectus muscle. MRM, medial rectus muscle

^a^The information above is related to the probands of CFEOM1 families indicated in Table 1

### DNA isolation

Peripheral blood samples were collected from affected individuals and available relatives for genomic DNA extraction, mutation analysis, and haplotype analysis. A volume of 4 ml venous blood sample was collected into ethylenediaminetetraacetic acid‑treated tubes. DNA extraction from peripheral blood leukocytes was performed using the DNA extraction kit (Qiagen, China) with standard protocols.

### Mutation analysis

Exons and flanking exon–intron boundaries of the *KIF21A* gene (38 exons) were amplified using polymerase chain reaction (PCR) analysis with the primers shown in Supplementary File 1. Briefly, PCR was performed in a 20 µl reaction volume with 2 µl each primer, 1 µl DNA, 10 µl buffer mix, and 7 µl ddH_2_O. All reagents used for PCR were purchased from Takara Bio, Inc. (Tokyo, Japan). The cycling profile was as follows: one cycle at 94˚C for 5 min, followed by 40 cycles at 94˚C for 45 s, 59˚C for 45 s, and 72˚C for 45 s, with a final cycle at 72˚C for 10 min. The PCR products were purified and used as templates for direct sequencing by the ABI 300 Genetic Analyzer (Perkin-Elmer, America). The sequencing results were analyzed using Seqman Pro 7.1.0 in Lasergene package (DNAStar, Madison, WI, USA). Variations were identified by aligning sequences with the reference sequences from the National Center for Biotechnology Information database (NCBI; https://www.ncbi.nlm.nih.gov/). Detected variations were further analyzed by cosegregation analysis in all available family members.

### Haplotype analysis

Based on the Genethon human genetic linkage map [21] in the National Center for Biotechnology Information, microsatellite markers with heterozygosity less than 0.75 but above 0.5 located within 2 Mb of *KIF21A* were selected. Four microsatellite markers D12S1692, D12S331, D12S1048, and D12S1668 were selected in these families, and the reference sequence of the microsatellite markers was obtained from the National Center for Biotechnology Information database (NCBI; https://www.ncbi.nlm.nih.gov/). The primers for amplifying the microsatellite markers were listed in Supplementary File 2. The details of the PCR procedure were the same as those described in mutation analysis. The PCR product was cloned into the TA cloning vector (Takara Bio Inc., Tokyo, Japan) and sequenced directly by the ABI 300 Genetic Analyzer (Perkin-Elmer, America). Family pedigrees and locus haplotypes were generated using the Cyrillic 2.1 program (Cyrillic Software, Wallingford, Oxford-shire, UK), and reconfirmed by observation.

### Mosaicism rate calculation

The mosaicism rate was defined as the proportion of presumed mosaicism family in total families. The overall mosaicism rate was calculated by combining our study with previous studies (Table 3). The 95% confidence interval (CI) of the pooled rate was estimated by the Clopper-Pearson method based on the exact binomial distribution.

**Table 3:** Genotype comparison of CFEOM1 patients in different studies

Sources	Country	Family	Mosaicism proportion^a^	*KIF21A* mutation site	Mutation rate^b^
[16]	USA	1	1 out of 1	c.1056C > G (p.D352E)	1 out of 1
[22]	Iran	1	0	c.2860C > T (p.R954W)	1 out of 1
[23]	India	1	0	c.2860C > T (p.R954W)	1 out of 1
[24]	China	1	0	c.2860C > T (p.R954W)	1 out of 1
[25]	China	1	0	c.2860C > T (p.R954W)	1 out of 1
[26]	China	1	1 out of 1	c.2860C > T (p.R954W)	1 out of 1
[27]	China	1	1 out of 1	c.2860C > T (p.R954W)	1 out of 1
[28]	China	1	0	c.2861G > A (p.R954Q)	1 out of 1
[29]	Saudi Arabia	1	1 out of 1	c.2861G > T (p.R954L)	1 out of 1
[15]	China	2	1 out of 2	c.2861G > A (p.R954Q)	1 out of 2
				c.3000_3002delTGA (p.D1001del)	1 out of 2
[30]	China	3	1 out of 3	c.2860C > T (p.R954W)	2 out of 2
[31]	China	3	0	c.2860C > T (p.R954W)	3 out of 3
[32]	Sweden, Turkey, France, Iran	4	1 out of 4	c.2860C > T (p.R954W)	4 out of 4
[17]	China	4	0	c.84C > G (p.C28W)	1 out of 4
				c.2860C > T (p.R954W)	2 out of 4
				Not found	1 out of 4
[33]	China	16	44%	c.2860C > T (p.R954W)	75%
				c.2861G > A (p.R954Q)	13%
				Not found	13%
[34]	USA, Spain, Turkey, Iran	16	63%	c.1067 T > C (p.M356T)	6%
				c.2830G > C (p.E944Q)	6%
				c.2860C > T (p.R954W)	31%
				c.2861G > A (p.R954Q)	13%
				c.2861G > T (p.R954L)	6%
				c.3022G > C (p.A1008P)	6%
				Not found	31%
[6]	USA, Canada, Spain, Turkey, Chile, Germany, Italy, Egypt, Netherlands, Venezuela, Australia	45	29%	c.1067 T > C (p.M356T)	4%
				c.2839A > G (p.M947V)	2%
				c.2840 T > C (p.M947T)	2%
				c.2840 T > G (p.M947R)	2%
				c.2860C > T (p.R954W)	71%
				c.2861G > A (p.R954Q)	13%
				c.3029 T > C (p.I1010T)	4%
This study	China	16	50%	c.2860C > T (p.R954W)	50%
				c.2861G > A (p.R954Q)	13%
				c.2861G > T (p.R954L)	13%
				Not found	25%
Overall	─	118	38.1%	c.84C > G (p.C28W)	0.8%
				c.1056C > G (p.D352E)	0.8%
				c.1067 T > C (p.M356T)	2.5%
				c.2830G > C (p.E944Q)	0.8%
				c.2839A > G (p.M947V)	0.8%
				c.2840 T > C (p.M947T)	0.8%
				c.2840 T > G (p.M947R)	0.8%
				c.2860C > T (p.R954W)	63.6%
				c.2861G > A (p.R954Q)	11.9%
				c.2861G > T (p.R954L)	3.4%
				c.3022G > C (p.A1008P)	0.8%
				c.3029 T > C (p.I1010T)	1.7%
				c.3000_3002delTGA (p.D1001del)	0.8%
				Not found	10.2%

^a^The proportion of presumed mosaicism family in the study

^b^The rate of family carry that mutation in the study

## Results

### Clinical findings

**Fig. 1:**
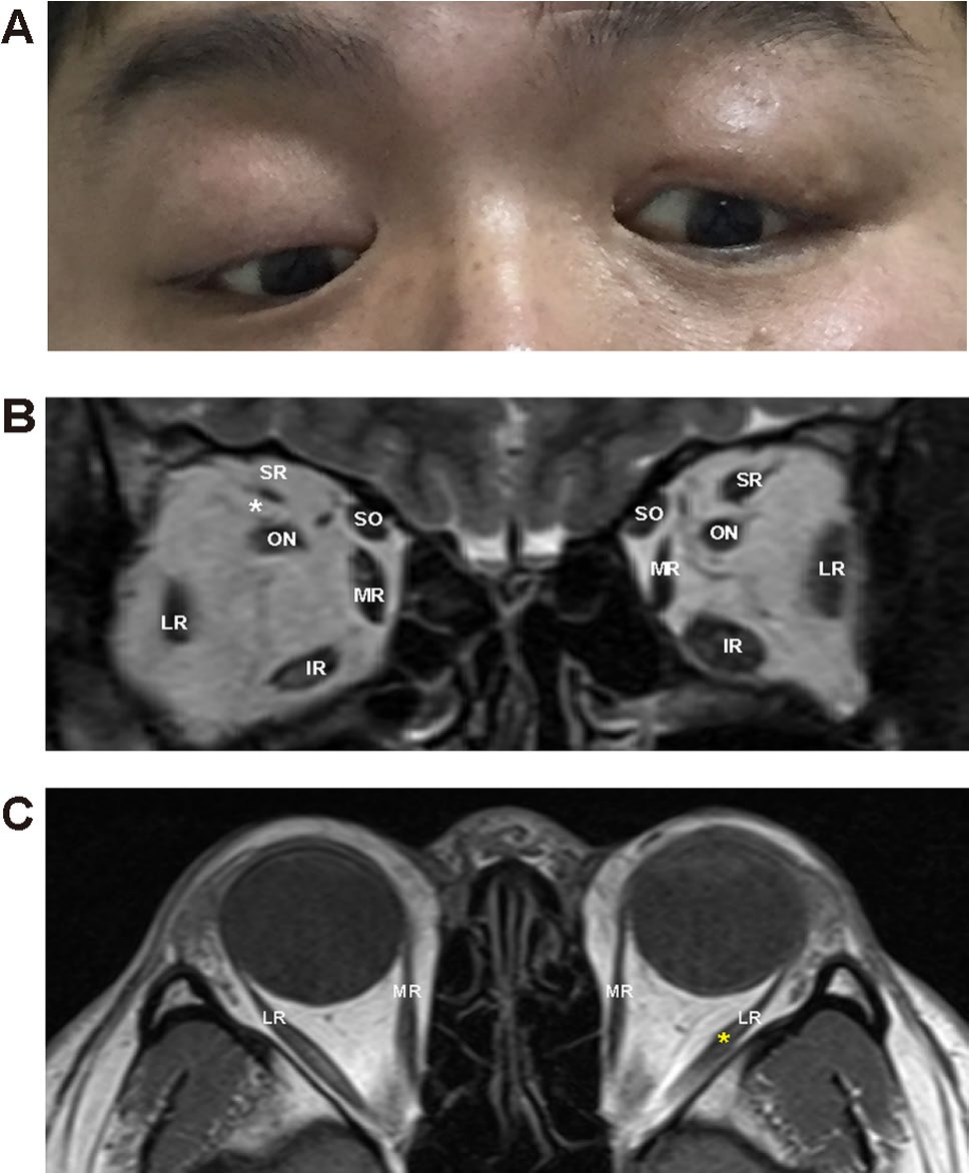
The eye position and MRI in Subject 02. **A** Eye position demonstrated ptosis, esotropia, and hypoplasia. **B** Coronal MRI showed atrophy of the left SR muscle (white asterisk). **C** Axial MRI showed the LR muscle of the left eye had a string-like configuration, which suggested muscle fibrosis the hypoplasia (yellow asterisk). MRI, magnetic resonance imaging; SR, superior rectus. LR, lateral rectus. MR, medial rectus. IR, inferior rectus. SO, superior oblique. ON, optic nerve

A total of 35 patients (18 males and 17 females from 16 families) were identified with classic CFEOM1 phenotypes, including bilateral congenital non-progressive ptosis, restricted horizontal eye movement, fixed infraduction, compensatory chin up head position, poor visual acuity, and stereopsis. Clinical data of each proband in 16 CFEOM1 families were summarized in Table 1. The MRI images of the affected individuals demonstrated hypoplasia of extraocular muscles in CFEOM1 typically (Fig. 1).

### Genetic analysis

**Fig. 2:**
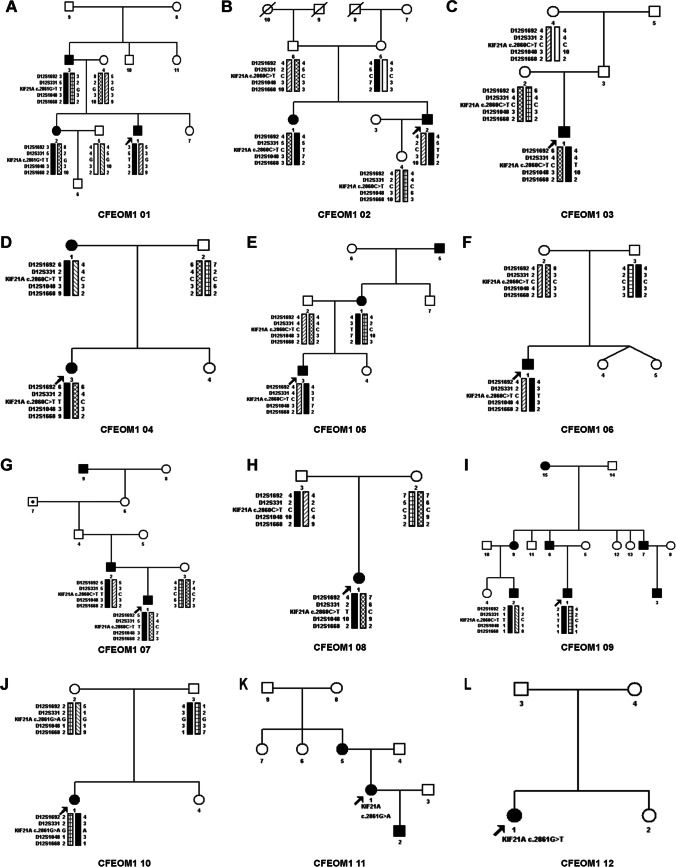
Pedigrees of CFEOM1 families identified with *KIF21A* mutations. **A**-**L** Genogram and haplotype of CFEOM1 family 01 to 12. Squares represent males, circles represent females, arrows indicate probands, and black symbols identify clinically affected individuals. Eight families (02, 03, 04, 05, 06, 07, 08, and 09) with c.2860C > T (p.R954W), two families (01 and 12) with c.2861G > T (p.R954L), and two families (10 and 11) with c.2861G > A (p.R954Q) were identified. *The palpebral fissure length*

Through mutation screening, we found three heterozygous mutations of the *KIF21A* gene in this study (Tables 1 and 3). *KIF21A* c.2860C > T (p.R954W) was found in eight families (CFEOM 1 family 02, 03, 04, 05, 06, 07, 08, and 09), c.2861G > T (p.R954L) was found in two families (CFEOM 1 family 01 and 12), and c.2861G > A (p.R954Q) was found in two families (CFEOM 1 family 10 and 11) (Fig. 2).

However, *KIF21A* mutations were not found in four families (CFEOM 1 family 13, 14, 15, and 16) (Supplementary File 3). A total of eight families (CFEOM1 family 02, 03, 06, 08, 10, 12, 14, 15) had affected individuals and asymptomatic parents without *KIF21A* mutation, which strongly suggested a high occurrence rate of germline mosaicism in CFEOM1 families. Interestingly by combining our results with those from the previous studies, we found c.2860C > T (p.R954W), c.2861G > T (p.R954L), and c.2861G > A (p.R954Q) presented in 63.6%, 3.4% and 11.9% CFEOM1 families, respectively. The overall mosaicism rate of CFEOM1 combined with these studies was 38.1% (95% confidence interval: 29.4% to 47.5%) (Table 3).

### Surgical management

Surgical plans were made individually according to the severity of ocular muscle paralysis and ptosis at the patient's informed preference. A total of six affected individuals underwent strabismus surgeries, and five underwent ptosis surgeries in this study (Table 2). Among six individuals who went strabismus surgeries, five of them underwent bilateral inferior rectus muscle recession, with or without inferior rectus muscle suspension to treat fixed infraduction. Some of the patients also underwent lateral/medial rectus muscle recession or rectus muscle transposition to treat exotropia or esotropia. After surgery, 2 out of 6 patients achieved orthotropia in primary position, and 4 out of 6 patients achieved microtropia, which implied that well-planned strabismus surgery was highly effective to improve ocular muscle movement in CFEOM1 patients.

**Fig. 3:**
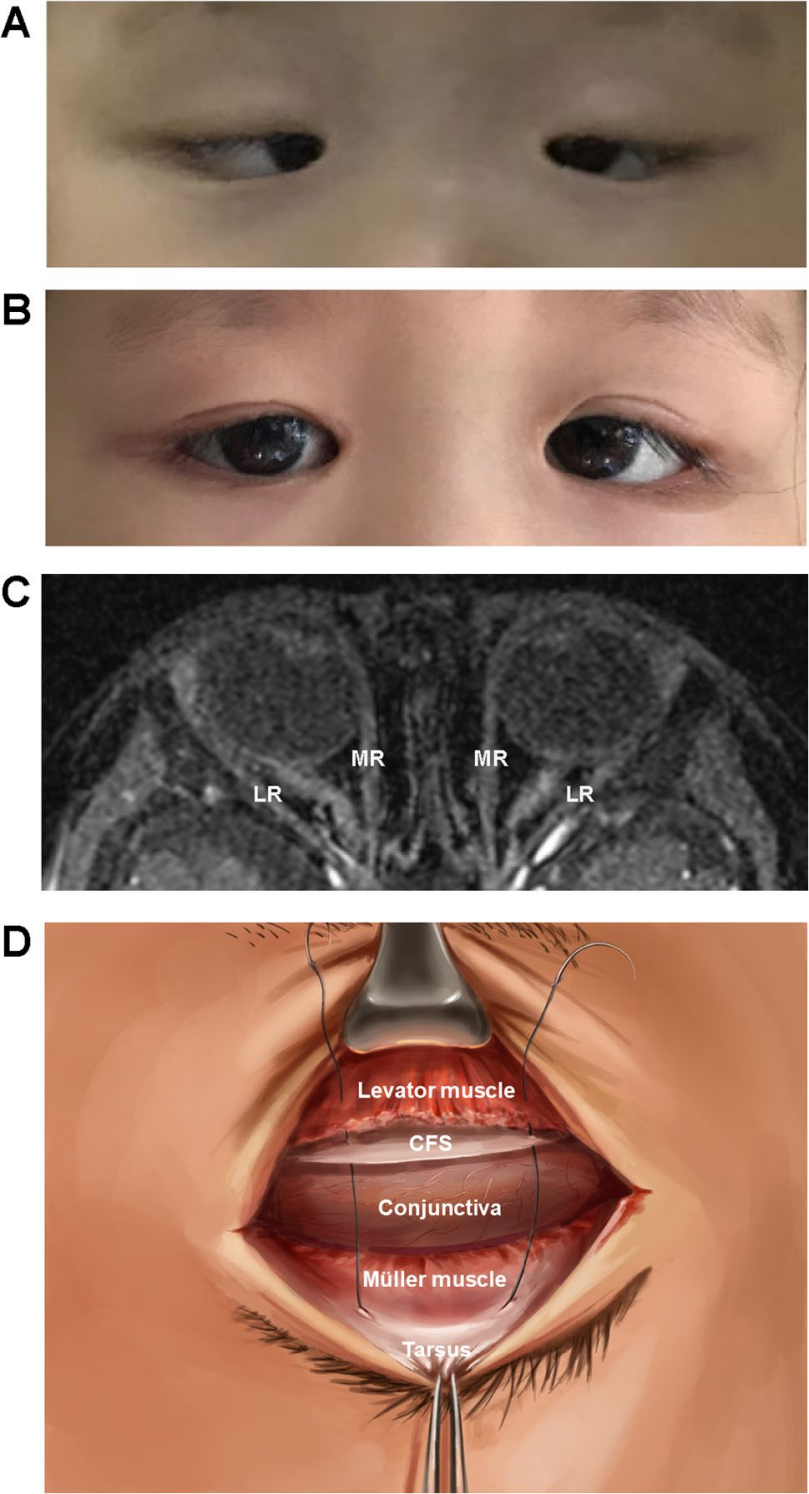
Effects and diagram of surgical management, and MRI in Subject 15. **A** Before ptosis and strabismus surgery, the individual was presented with severe ptosis, fixed infraduction, and esotropia. **B** After surgery, she got an excellent prognosis with bilateral PFH improvement and microtropia. **C** MRI showed slight atrophy of bilateral LR, compared to MR. **D** Diagram of modified levator muscle complex suspension. MRI, magnetic resonance imaging; SR, superior rectus. LR, lateral rectus. MR, medial rectus. IR, inferior rectus. SO, superior oblique. ON, optic nerve. CFS, combined fascia sheath

Five individuals went through ptosis surgery, including three of them who had levator palpebrae superioris muscle shortening or frontal muscle flap suspension before visiting our department, which resulted in under-correction. The other two individuals, Subjects 08 and 15 with severe congenital ptosis, achieved an excellent prognosis via a novel ptosis procedure. It was a modified levator muscle complex suspension, which is performed by combined fascia sheath and levator muscle complex suspension with Müller muscle preservation [20] (Table 2, Fig. 3). Upon examination, bilateral blepharoptosis was observed in both individuals. Before surgery, both patients’ right and left palpebral fissures were 3 and 4 mm, respectively. They both have 1–2 mm myodynamia of the levator palpebrae muscle. They underwent surgery for the correction of bilateral ptosis and achieved a good result without any complications. Subject 15 also went through strabismus surgery, with bilateral lateral rectus muscle recession (6 mm) and bilateral inferior rectus muscle recession (8 mm).

## Discussion

In our study, we found three *KIF21A* mutations in 16 CFEOM1 families, including c.2860C > T (p.R954W) in eight families, c.2861G > T (p.R954L) in two families, and c.2861G > A (p.R954Q) in two families. Taken together with previous studies (Table 3), a total of 118 CFEOM1 families were involved in genetic analysis. Among these families, *KIF21A* c.2860C > T (p.R954W) (63.6%), c.2861G > A (p.R954Q) (11.9%), c.2861G > T (p.R954L) (3.4%) were the most commonly identified mutations, suggesting R954 mutations served as hotspot mutation of *KIF21A*. Therefore, R954 mutations could be screened first in the etiological investigation of CFEOM1 individuals in the future, instead of conducting experiments on whole exons of *KIF21A* for higher efficacy.

Among the three types of CFEOMs, the large majority of CFEOM1 and a few CFEOM3 are caused by mutations in the *KIF21A* gene, while CFEOM2 is associated with mutations in *PHOX2A* gene [30, 34]. *KIF21A* gene is mainly expressed in the brain, heart, skeletal muscle, and kidney, located in axons and dendrites in neurons [35]. This gene contains 38 exons and encodes KIF21A, a member of the KIF4 subfamily of kinesin-like motor protein consisting of 1674 amino acids. The main function of KIF21A is to participate in transporting intracellular substances for axon guidance, in a microtubule-dependent way [36].

KIF21A consists of three segments, an N-terminal motor region, a coiled-coil stalk region, and a C-terminal WD40 repeat region [37]. To date, a total of 12 different missense mutations and one deletion mutation have been detected in CFEOM1 individuals [15–17]. Three *KIF21A* mutations, c.84C > G (p.C28W), c.1056C > G (p.D352E), and c.1067 T > C (p.M356T), are located in the N-terminal motor region, which interacts with the microtubule track [6]. The remaining ten *KIF21A* mutations, c.2830G > C (p.E944Q), c.2839A > G (p.M947V), c.2840 T > C (p.M947T), c.2840 T > G (p.M947R), c.2860C > T (p.R954W), c.2861G > T (p.R954L), c.2861G > A (p.R954Q), c.3022G > C (p.A1008P), c.3029 T > C (p.I1010T), and c.3000_3002delTGA (p. D1001del), are all located in the third domain of coiled-coil region. Mutations in this region may affect the structural integrity and the autoinhibitory binding interface, hence reducing motor domain affinity [34]. The mutations in the WD40 repeat region haven’t been reported in CFEOM1 individuals, which is a putative cargo-binding region [38].

R954 is located in the third domain of the coiled-coil domain along with other *KIF21A* mutation sites and is highly conserved during evolution [34]. The replacement of the positively charged arginine residue with other amino acids (tryptophan, glutamine, or leucine) would seriously affect the function of the encoded protein, resulting in abnormalities in the encoded product. The substances required for the development of No. III cranial nerve motor axon, neuromuscular junctions, and innervated extraocular muscles were restricted, resulting in multiple extraocular muscle dysfunction.

As seen from the established *Kif21a* R954W knock-in mice model, the mice presented the same phenotypes (ptosis and restricted upgaze reflect) as CFEOM1 patients [39]. *Kif21a*^*KI*^ mice showed lower muscle fiber density in superior rectus muscle and inferior rectus muscle, compared with the wild-type littermates, which might result from abnormal oculomotor nerve innervation [39]. The study also demonstrated that interaction of the third coiled-coil region and motor was vital to Kif21a-microtubule association. Mutations in the motor and stalk region could lead to Kif21a accumulation and disrupt the normal interaction [39]. Another study also identified KIF21A as an inhibitor of microtubule growth in the cell cortex, restricting microtubule growth and organizing microtubule arrays at the cell edge in vivo [40]. These studies provided some evidence of the role of *KIF21A* in autoinhibitory, but the detailed mechanisms still need further research.

Germline mosaicism is a mutation that occurs barely in the gonads (ovary and testis) of the parents and can be inherited by their offspring [41]. A total of 8 families in our study were affected individuals with asymptomatic parents without *KIF21A* mutations, therefore the presumed mosaicism rate was 50% (8/16). Along with previous studies, 45 such families were identified from 118 CFEOM1 families, and the presumed mosaicism rate was 38.1%. As CFEOM1 and CFEOM3 were inherited in autosomal dominant patterns, whereas CFEOM2 was an autosomal recessive pattern, germline mosaicism of CFEOM1 could mimic the recessive inheritance of CFEOM2, which needs to be carefully investigated in genetic counseling and clinical diagnosis [29].

In concern of the management of CFEOM1 individuals, it was recommended to perform strabismus surgery when necessary. Extraocular muscle recession and suspension surgery were recommended rather than resection, because of the extraocular muscle restriction in CFEOM1 individuals [42]. Ptosis surgery is not considered as a routine way for it may increase the risk of exposed keratitis in CFEOM1 patients, especially in those who without Bell’s phenomenon. Frontalis muscle suspension and levator palpebrae superioris muscle shortening is the traditional procedure to treat ptosis [43]. However, it was related to complications including undercorrection, lagophthalmos, infection, and raised eyebrow [43–45]. Three individuals in this study underwent traditional ptosis surgery before visiting our department, and was presented with undercorrection. Two girls received the modified levator muscle complex suspension, a novel surgical technique. Through the anterior approach, Müller's muscle was preserved, meanwhile, the upper tarsus was suspended to combined fascia sheath and levator muscle complex [20]. After surgery, the two girls got an excellent prognosis without keratitis, and their chin-up symptoms improved as well, suggesting this novel approach might avoid keratitis and have high effectiveness in correcting eyelid abnormality.

This study has several strengths. First, this was one of the largest genetic studies of CFEOM1 patients in China. Second, our study reported the germline mosaicism rate and mutation rate using cumulative evidence from the literature, presumed the universality of germline mosaicism, and identified hotspot mutations in CFEOM1 families. Third, a novel procedure of modified levator muscle complex suspension was applied to treat CFEOM1 with high safety and efficacy.

Our study also has some limitations. First, like all other population-based genetic studies, it plays a limited role in investigating detailed disease mechanisms. However, it can act as a foundation for future functional studies at cellular and animal levels. Second, a small portion of families (25%, 4/16) had no *KIF21A* mutations found. Thirdly, the mosaicism rate was based on the assumption in our study, because participants disagreed to provide sperm or ovum samples. Fourth, the number of CFEOM1 patients eligible for modified levator muscle complex suspension was small, and more clinical practice is required to gather more evidence for its effectiveness and safety.

In this study, mutation screening suggested the hotspot mutations of CFEOM1 families are c.2860C > T (p.R954W) c.2861G > A (p.R954Q), and c.2861G > T (p.R954L). Future studies should prioritize screening for these mutations among CFEOM1 patients. Patients with CFEOM1 frequently exhibit chromosomal mosaicism, which must be carefully taken into account during clinical diagnosis. This study also suggests that the highly effective and safe modified levator muscle complex suspension procedure for treating ptosis may provide physicians with a novel option for treating CFEOM1.

## Supplementary Information

Below is the link to the electronic supplementary material.

Supplementary file1 (DOCX 21 KB)

Supplementary file2 (DOCX 17 KB)

Supplementary file3 (DOCX 82 KB)
