# Supplementary material for: Phenotype, genotype, and management of congenital fibrosis of extraocular muscles type 1 in 16 Chinese families
Source: Graefes Arch Clin Exp Ophthalmol. 2022 Sep 23;261(3):879–89. doi: 10.1007/s00417-022-05830-3 (PMC9988770; doi:10.1007/s00417-022-05830-3)
Supplement: Supplementary file 1 — Supplementary file1 (DOCX 21 KB) [file 417_2022_5830_MOESM1_ESM.docx]

**Supplementary File 1** Primers used for sequencing *KIF21A* exons and flanking exon-intron boundaries.

| Primer Name | Primer 5’-3’ |
| --- | --- |
| *KIF21A*-E1_Forward | gaatcacctcctcctcttgct |
| *KIF21A*-E1_Reverse | gactcactgcctcagtttcctc |
| *KIF21A*-E2_ Forward | ttggccaagagtttttaagtcc |
| *KIF21A*-E2_ Reverse | aaaaatgaaagcgcaactgaat |
| *KIF21A*-E3_ Forward | CAGCAAGAGCAGATCTACATTCA |
| *KIF21A*-E3_ Reverse | caaagcactatacttgaagccaac |
| *KIF21A*-E4_ Forward | gcctcattcattttaatgtgttatttt |
| *KIF21A*-E4_ Reverse | agcagattatcagcaaagctca |
| *KIF21A*-E5_ Forward | tccatctcaaaaagtgcctgta |
| *KIF21A*-E5_ Reverse | caaaatcttacatggctgacca |
| *KIF21A*-E6_ Forward | gccagcagacatttttgaattt |
| *KIF21A*-E6_ Reverse | gaggagattggagattcagtgc |
| *KIF21A*-E7_ Forward | aaattttcagtgacattggttacaa |
| *KIF21A*-E7_ Reverse | cctcatcctattagtttgctttgc |
| *KIF21A*-E8_ Forward | gtcttcaagtacaggggctttt |
| *KIF21A*-E8_ Reverse | agaaagtgccagccttagatgt |
| *KIF21A*-E9_ Forward | tgaaaacgctcttggttagaga |
| *KIF21A*-E9_ Reverse | tggcatttctatttcaactatgc |
| *KIF21A*-E10_ Forward | tcatattatcttgtggtctgctca |
| *KIF21A*-E10_ Reverse | atgggaagagaaaggaggaaaa |
| *KIF21A*-E11_ Forward | cagagctgtacacttaagaaatgg |
| *KIF21A*-E11_ Reverse | ttgtagtttcagctaacatagtgattt |
| *KIF21A*-E12_ Forward | ttcaagctcatgtatcatttttacc |
| *KIF21A*-E12_ Reverse | gcaggggttttagggttatttt |
| *KIF21A*-E13_ Forward | ccaacatttaaagtttgttttcaca |
| *KIF21A*-E13_ Reverse | atcaaccaagaacaaccaaacc |
| *KIF21A*-E14_ Forward | atgagggagtcagtttgttttg |
| *KIF21A*-E14_ Reverse | aggtcagtggtcacagaagttt |
| *KIF21A*-E15_ Forward | caccttttggttgttggttttt |
| *KIF21A*-E15_ Reverse | aacccattttgaagatttagcttg |
| *KIF21A*-E16_ Forward | tcttctgagatgtgggtgtttg |
| *KIF21A*-E16_ Reverse | aggcttggatgaggaatgacta |
| *KIF21A*-E17_ Forward | tgaatgttgtgacagagtaatttcaa |
| *KIF21A*-E17_ Reverse | tcatctggaaaataagcctcaa |
| *KIF21A*-E18_ Forward | actcatagttcccaggggtatg |
| *KIF21A*-E18_ Reverse | CGAAGAGCCGTAACctgaaat |
| *KIF21A*-E19_ Forward | tttatagCATCAACTTAGACTTCTGGA |
| *KIF21A*-E19_ Reverse | gattgtgcactgccaaataatg |
| *KIF21A*-E20_ Forward | ggcagtgcacaatcaaataagt |
| *KIF21A*-E20_ Reverse | ttgagaaagcaggttggatttt |
| *KIF21A*-E21_ Forward | caattttgttccctgggtaaat |
| *KIF21A*-E21_ Reverse | aaaaactgaaagtgctttgaaca |
| *KIF21A*-E22_ Forward | tgcacttatctgagcatctcatt |
| *KIF21A*-E22_ Reverse | ttcctgactctaaagaaaggtaaaa |
| *KIF21A*-E23_ Forward | tgtttgatacagatctttgtgtgg |
| *KIF21A*-E23_ Reverse | gccaaaatgtacacatgcaaa |
| *KIF21A*-E24_ Forward | tgtgggagtttttaacattgga |
| *KIF21A*-E24_ Reverse | gaggcacaactggaactcagat |
| *KIF21A*-E25_ Forward | gaacacacaatcaaaggagagttt |
| *KIF21A*-E25_ Reverse | ttcactaggccattagattgaaaa |
| *KIF21A*-E26_ Forward | tttttgtagtcgttgatatagaggag |
| *KIF21A*-E26_ Reverse | gctggaattacaggcatgag |
| *KIF21A*-E27_ Forward | tatgtttggcacacctaggaaa |
| *KIF21A*-E27_ Reverse | gccaaaagaaaagaagttagtgtagc |
| *KIF21A*-E28_ Forward | caagtaataatctttctgaggttcca |
| *KIF21A*-E28_ Reverse | agccaaaagctgacttgactgt |
| *KIF21A*-E29_ Forward | aaaagagcatgtttcttataacttttc |
| *KIF21A*-E29_ Reverse | ttttctctaattttggctgcat |
| *KIF21A*-E30_ Forward | gaaaattttagcccccaaagtc |
| *KIF21A*-E30_ Reverse | tgcatttatatgatttacaacaagtga |
| *KIF21A*-E31_ Forward | ctgggggtccttaacttctctt |
| *KIF21A*-E31_ Reverse | aaacatacagccatggtatttctc |
| *KIF21A*-E32_ Forward | tgcttaaaagagagcagtctgg |
| *KIF21A*-E32_ Reverse | tccgattctttctggtcttaaa |
| *KIF21A*-E33_ Forward | ttttcatttccttcccatagaa |
| *KIF21A*-E33_ Reverse | tgcatttatagtcctctcttttctt |
| *KIF21A*-E34_ Forward | tcaaggaattctaagccaaacc |
| *KIF21A*-E34_ Reverse | ttttacattaacttctggggtca |
| *KIF21A*-E35_ Forward | cccaagatcccatctctaaaca |
| *KIF21A*-E35_ Reverse | gaaaaagcaaagactagaaaggaaaa |
| *KIF21A*-E36_ Forward | aaaagaaaacatttaacaaaccagaaa |
| *KIF21A*-E36_ Reverse | ttcccttctcccagtgaaga |
| *KIF21A*-E37_ Forward | ctttctccagccaattccaa |
| *KIF21A*-E37_ Reverse | tgcccacatgtacatcagaaa |
| *KIF21A*-E38_ Forward | cttggatttcttgaagacctatg |
| *KIF21A*-E38_ Reverse | aagcacacaggatagtacacaattt |
