# Supplementary material for: Phenotype, genotype, and management of congenital fibrosis of extraocular muscles type 1 in 16 Chinese families
Source: Graefes Arch Clin Exp Ophthalmol. 2022 Sep 23;261(3):879–89. doi: 10.1007/s00417-022-05830-3 (PMC9988770; doi:10.1007/s00417-022-05830-3)
Supplement: Supplementary file 2 — Supplementary file2 (DOCX 17 KB) [file 417_2022_5830_MOESM2_ESM.docx]

**Supplementary File 2** Primers used for microsatellite markers D12S1692, D12S331, D12S1048, and D12S1668.

| Primer Name | Primer 5’-3’ |
| --- | --- |
| D12S1692_Forward | agtccgggcaacagagcaa |
| D12S1692_Reverse | gcaatttcagacttctcccgaat |
| D12S331_ Forward | agctattttaacatttattcatctcc |
| D12S331_ Reverse | agctnagacatgtaagagagnaag |
| D12S1048_ Forward | tggtctgcttaggtccctttt |
| D12S1048_ Reverse | caaggaaccaaggagtggaa |
| D12S1668_ Forward | ttagccaggtgtggtggt |
| D12S1668_ Reverse | caatagagattagtgtcacctgatttt |
