# Supplementary material for: Phenotype, genotype, and management of congenital fibrosis of extraocular muscles type 1 in 16 Chinese families
Source: Graefes Arch Clin Exp Ophthalmol. 2022 Sep 23;261(3):879–89. doi: 10.1007/s00417-022-05830-3 (PMC9988770; doi:10.1007/s00417-022-05830-3)

**Supplementary File 3** Pedigrees of CFEOM1 families not identified with *KIF21A* mutations. (A-D) Genogram of CFEOM1 family 13 to 16. Squares represent males, circles represent females, arrows indicate probands, and black symbols denote clinically affected individuals.


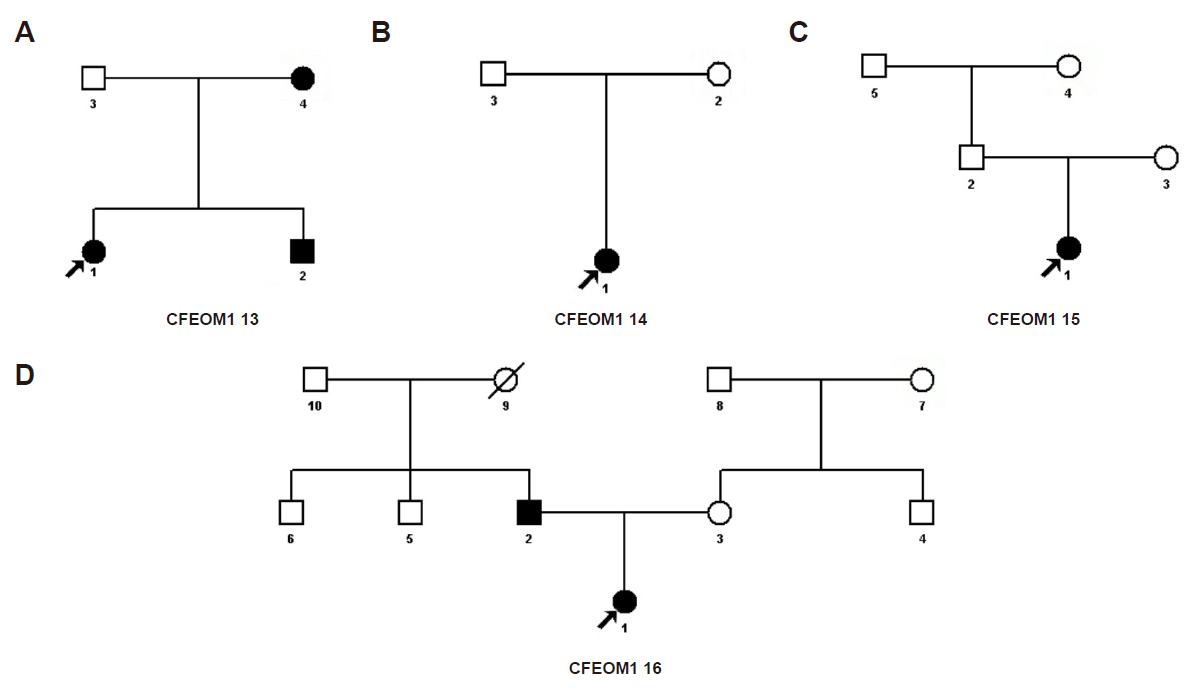

Supplement: Supplementary file 3 — Supplementary file3 (DOCX 82 KB) [file 417_2022_5830_MOESM3_ESM.docx]
